# Perfluoroalkyl Substance Exposure and the BDNF Pathway in the Placental Trophoblast

**DOI:** 10.3389/fendo.2021.694885

**Published:** 2021-07-28

**Authors:** Melissa J. Marchese, Shuman Li, Bin Liu, Jun J. Zhang, Liping Feng

**Affiliations:** ^1^Department of Obstetrics and Gynecology, Duke University, Durham, NC, United States; ^2^Ministry of Education-Shanghai Key Laboratory of Children’s Environmental Health, School of Public Health, Shanghai Jiao-Tong University School of Medicine, Shanghai, China

**Keywords:** Perfluoroalkyl substances, BDNF, TrkB, placenta, trophoblast

## Abstract

**Background:**

Per- and polyfluoroalkyl substances (PFAS) are persistent organic pollutants that have become globally ubiquitous in humans and the environment. *In utero* PFAS exposure is associated with neurodevelopmental effects; however, the mechanism is poorly understood. Brain-derived neurotrophic factor (BDNF) signaling is critical to fetal neurodevelopment during pregnancy and maintains important regulatory roles later in life. This study aims to characterize placental BDNF signaling and investigate whether PFAS exposure disrupts the signaling pathway in placental trophoblast cells.

**Methods:**

The expression and localization of BDNF receptors–p75^NTR^ and TrkB–in first trimester and term human placentas and trophoblast cells were investigated by immunofluorescence staining. To assess the effects of PFAS exposure on the BDNF pathway, BeWo cells were treated with PFAS mixtures that mimicked blood levels in a highly exposed population and major PFAS compounds in the mixture at 0.01, 0.1, 1, and 10 µM concentrations. Changes in pro-BDNF levels and phosphorylation of TrkB receptors were examined by Western blot.

**Results:**

In first trimester human placentas, TrkB and p75^NTR^ receptors were primarily localized to syncytiotrophoblast and cytotrophoblast cells. At term, TrkB and p75^NTR^ receptors were primarily observed in the placental villous stroma. TrkB receptor staining in trophoblasts was reduced at term, while p75^NTR^ receptor staining was negative. TrkB receptors were confined to the nuclear and perinuclear spaces, and phosphorylation occurred at the Tyr816 residue in BeWo cells. Exposure to PFOS, PFOA, PFBS, and the six-PFAS mixture did not significantly affect BDNF levels or activation (phosphorylation) of TrkB. Treating cells with 1 μM and 10 μM of PFNA resulted in increased TrkB phosphorylation compared to unexposed controls, but BDNF levels were unchanged.

**Conclusions:**

BDNF receptors are present in different regions of human placental villi, indicating diverse functions of BDNF signaling in placental development. Our findings suggest that the BDNF pathway in placental trophoblast cells is not disrupted by exposures to PFOS, PFOA, PFBS, and a PFAS mixture, but may be affected by PFNA exposures. Further investigation is needed on how PFAS affects other critical signaling pathways during fetal neurodevelopment.

## Introduction

Per- and polyfluoroalkyl substances (PFAS) are synthetic carbon fluorine compounds used in industry and commerce since the 1940s (1). The stability and surfactant properties exhibited by PFAS have led to widespread use in products from stain-resistant textiles to firefighting foam—making them ubiquitous in the environment (2, 3). Biomonitoring data have demonstrated global accumulation in humans and biota *via* contaminated drinking water, food and its packaging, outdoor air, house dust, and soil (4–6). Previous studies estimate over 95% of Americans to have detectable levels of PFAS in their blood, but certain communities face disproportionate exposure risk (7–9). One such affected population is in Pittsboro, North Carolina, where sum PFAS concentrations up to 1,076 ng/L and 270.8 ng/L were measured at drinking water intakes and in finished household drinking water, respectively.^1^ These numbers are over 30 times higher than those in the neighboring city of Durham and are strikingly higher than surrounding cities.^1^ The blood levels of PFAS are between two and four times higher in Pittsboro residents than the general United States population.^2^

Concern surrounding PFAS has risen among affected North Carolina residents due to the potential deleterious human health effects associated with exposure. A growing body of robust laboratory and epidemiologic data link PFAS to increased risk of cancer, immune system dysfunction, poor antibody response to vaccines, hormone disruption, kidney disease, thyroid disorders, high cholesterol, pregnancy-induced hypertension, preeclampsia, asthma, and decreased fertility (10). Fetuses are particularly vulnerable to PFAS exposure because PFAS readily cross the placental barrier and are transferred to developing babies (11–13). Early-life exposure to various PFAS compounds is associated with an array of developmental and neurobehavioral impacts. Previous early childhood studies observed associations between PFAS exposure and delays in gross motor development (14), worsened visual motor abilities (15), lower IQ test scores (16), externalizing behavioral difficulties (17–19), poorer executive functioning (20), neuropsychological developmental problems (21), and attention deficit hyperactivity disorder (ADHD) and related diagnostic symptoms (19, 22, 23). Other observational studies of child neurobehavior report mixed or null associations (24–29). Despite the effects of PFAS on human development gaining more attention, a consensus on the neurodevelopmental consequences of exposure has yet to be reached and further investigation is necessary.

Although the mechanism of neurotoxicity remains unclear, one pathway that may be implicated is the production of brain-derived neurotrophic factor (BDNF). BDNF is essential to mammalian nervous system development and function (30). BDNF primarily interacts with two receptors, p75^NTR^ and tropomyosin sensitive receptor kinase B (TrkB) (31, 32). Abnormalities in the BDNF pathway have been linked to psychiatric disorders such as ADHD, schizophrenia, major depression, autism, bipolar disorder, Alzheimer’s, and Parkinson’s diseases (1, 5, 33–38). Reduced expression of BDNF has been shown to impair working memory, reduce volume of the cerebellum and hippocampus, and diminish cognitive ability (39). Neurotrophins like BDNF play a role in both prenatal and postnatal brain development and may offer neuroprotective effects (40–42). BDNF supports differentiation of neuronal cell types and influences synaptic properties in the peripheral and central nervous systems during early development (41, 43, 44). During pregnancy, the majority of BDNF is derived from either the mother or the placenta and regulates fetal and placental development (45–48). BDNF contributes to fetal growth by promoting survival, proliferation, migration, and differentiation of cytotrophoblasts in addition to angiogenesis, vessel stabilization, regulation of growth factors, and control of energy homeostasis (48–51). Changes in the BDNF pathway may lead to abnormal placental development and pregnancy complications such as preeclampsia (52, 53). However, the characteristics of the BDNF expression in the human placenta are poorly understood. *BDNF* is a plastic gene whose expression is highly responsive to external stimuli and environmental exposures (30, 35, 37, 54). Significant PFAS-attributable alterations in BDNF expression have been observed in several animal model and cell studies, but conclusions are largely inconsistent (55–59).

This study aims to characterize the BDNF pathway in human placental tissues and trophoblast cell lines. Further, we seek to investigate how the BDNF pathway in placental trophoblast cells is affected by exposures to a PFAS mixture—which models the exposure of Pittsboro, North Carolina residents (based on blood levels)—and major PFAS compounds in this mixture.

## Materials and Methods

### Chemicals and Reagents

Potassium nonafluoro-1-butanesulfonate (K+PFBS, CAS No. 29420-49-3), tridecafluorohexane-1-sulfonic acid potassium salt (K+PFHxS), and heptadecafluorooatanesulfonic acid potassium salt (K+PFOS) were purchased from Sigma-Aldrich (St. Louis, MO). Oerfluorohexanoic acid (PFHxA), perfluoro-n-octanoic acid (PFOA), perfluorononanoic acid (PFNA), and perfluorodecanoic acid (PFDA) were purchased from Synquest Laboratories (Alachua, FL). Product purity is above 95%. Stock solutions of PFAS at 10, 1, 0.1, 0.01 mM were prepared by dissolving PFAS compounds in ultrapure distilled water. A PFAS mixture was made based on the PFAS levels in blood samples collected from people living in Pittsboro, NC, in 2019 (**Table 1**).^3^ Forskolin (Wako Chemicals, Japan) stock solution was reconstituted in DMSO at 80 mM.

**Table 1:** PFAS mixture (ng/g) administered to cell cultures based on levels in blood samples collected from residents of Pittsboro, North Carolina.

PFOS	12.10
PFOA	7.00
PFHxS	3.10
PFNA	1.80
PFDA	0.75
PFHxA	0.60
Total	25.35

### Cell Culture and PFAS Treatments

An immortalized human choriocarcinoma cell line (BeWo cells, gifted by Dr. Sallie Permar, Duke University, Durham, North Carolina) was cultured in Dulbecco’s Modified Eagle Medium-Hams/F12 (DMEM/F12) media supplemented with 10% Fetal Bovine Serum (FBS) and 1x antibiotic-antimycotic solution (Thermo Fisher Scientific). Cell culture and treatment conditions are described in our previous publications (60, 61). In short, after 60-70% confluency was reached, cells were treated for 48 hours with DMSO (1:2000 dilution) or 40 μM forskolin (FSK) to induce cell fusion, thereby modeling syncytialization of cytotrophoblasts (CTB) during placental development. Culture media supplemented with fresh 40 μM FSK was changed every 24 hours to ensure complete cell fusion. BeWo cells with and without FSK treatments were then treated with individual PFAS compounds at 0, 0.01, 0.1, 1, and 10 μM concentrations or the PFAS mixture (1:1, 1:10, 1:50, 1:100 dilutions; **Table 1**) for 24 hours. These doses were chosen for cellular function studies based on human exposure levels.^3^ To ensure the effects on cellular functions are not due to cytotoxicity, we used doses at least 10 times lower than LD10 (61). The following methods for immunofluorescence staining and Western blot were performed as described in Marinello et al. (62).

### Immunofluorescence Staining of Placental Tissues

First trimester placental tissues were collected from elective pregnancy termination patients without notifiable complications at 8-10 gestational weeks. The study protocol was approved by the Ethics Committees of Xinhua Hospital affiliated with Shanghai Jiao Tong University School of Medicine (IRB# XHEC-C-2018-089) with the consent waiver to obtain de-identified tissue that was not to be used for clinical purposes. Term placental tissues were collected under the Duke University Institutional Review Board approval (IRB# PRO00014627) with the consent waiver to obtain de-identified tissue that was not to be used for clinical purposes. The placentas were collected from women who underwent planned cesarean delivery at term (39 to 40 gestational weeks), without labor and current or previous pregnancy complications.

Sections of placental tissues were fixed and paraffin embedded. Tissue slides were deparaffinized with xylene followed by graded rehydration in ethanol and distilled water. Subsequently, the sections were subjected to heat-induced epitope retrieval by heating in antigen unmarking solution (Vector Laboratories, Inc, Burlingame, California) preheated to boiling for 20 minutes, followed by a 20-minute cool-down period at room temperature. Slides were permeabilized and blocked with 1% BSA, 10% normal goat serum and 0.01% tween-20 in PBS for 60 min at room temperature. After blocking, the slides were incubated with primary antibodies in blocking buffer overnight at 4°C in humidified chambers. Primary rabbit anti-human syndecan 1 (SDC-1) antibody (Sigma, catalog no. HPA006185), mouse anti-human TrkB antibody (R & D System, cat#: MAB397), or p75NTR antibody (R & D System, cat#: MAB3671) was used at a 1:200 dilution. Goat anti-rabbit Alexa Fluo 488 secondary antibody and Goat anti-mouse Alexa Fluo 594 secondary antibody (Life Technologies, Carlsbad, CA) were used at 1:300 dilution. Nuclei were stained using NucBlue Fixed Cell ReadyProbes Reagent (DAPI, Invitrogen). Slides were mounted using ProLong Diamond Antifade Mountant (Invitrogen) and examined with a Leica SP5 inverted confocal fluorescence microscope.

### Immunofluorescence Staining of BeWo Cells

BeWo cells were seeded at a density of 2 x 10^4^ cells per well in glass chamber slides (ibidi GmbH, Germany) then incubated for 24 hours. Cells were fixed with cold methanol for 10 minutes and blocked with 1% BSA, 5% goat serum, and 0.1% Tween-20 in PBS for 1 hour at room temperature. Next, cells were incubated at 4°C overnight with primary antibodies in humidified chambers. 1:100 dilutions of primary rabbit anti-human TrkB antibodies (Abcam, cat#: ab18987) and p75NTR antibodies (Abcam, cat#52987) were used. Anti-rabbit IgG antibodies served as a negative control (R & D system, Minneapolis, MN). 1:300 dilutions of goat anti-rabbit secondary antibodies and Alexa Fluo 594 (Life Technologies, Carlsbad, CA) were used. F-actin was stained using fluorescein Phalloidin (Invitrogen, cat# F432) at 1:300 dilution. Nuclei were stained using NucBlue Fixed Cell ReadyProbes Reagent (DAPI, Invitrogen). Slides were mounted using ProLong Diamond Antifade Mountant (Invitrogen) and examined with a Leica SP5 inverted confocal fluorescence microscope.

### Cell Viability Assay

BeWo cells were seeded at a density of 1 x 10^4^ cells per well in 96-well plates and incubated for 24 hours. The cells were then treated with each individual PFAS compound at 0, 0.01, 0.1, 1, 10 μM or a PFAS mixture (**Table 1**) at 1:1, 1:10, 1:50, 1:100 dilutions in quadruplicate for 24 hours. Cell viability was measured using the CellTiter 96^®^ AQueous One Solution Cell Proliferation Assay kit as instructed by the manufacturer instructions (G5421; Promega, Madison, WI). The optical density at 490 nm (OD490) was used to quantitatively compare cell viability between treatments after incubating the cells with MTS reagents for 4 hours. These experiments were repeated five times (N=5).

### Western Blot

To harvest cell lysates after treatment, we used RIPA buffer (Sigma Aldrich, St. Louis, MO) with the complete mini-protease inhibitor cocktail (Roche, Mannheim, Germany). Protein concentrations were determined with the Bradford assay (Bio-Rad Laboratories, Hercules, CA). 25μg of total protein samples were loaded onto 10% sodium dodecyl sulfate polyacrylamide gels, separated, and transferred to a polyvinylidene difluoride membrane. The membranes were blocked with 5% milk in Tris-Buffered Saline and Tween-20 (TBST) buffer. Membranes were probed in blocking buffer at 4°C with primary antibodies overnight. The following primary antibodies were used in the experiments: rabbit anti-human TrkB antibody (Abcam, cat#: ab18987), phosphor-TrkB (Tyr816) antibody (Cell Signaling Technology, cat#4168), and rabbit anti-human pro-BDNF antibody (Invitrogen, cat# PA1-18360) at 1:1000 dilution; rabbit anti-human GAPDH antibody at 1:20 000 dilution. The secondary antibody was diluted at a ratio of 1:2000. To visualize and photograph the membranes, we used the ChemiDoc MP Imaging System with Image Lab Software (Bio-Rad, Berkeley, CA). Band intensity was quantified with ImageJ (NIH, Bethesda, MD). Data were normalized to the internal control (GAPDH) and are presented as ratios. Experiments were repeated four times (N=4).

### Statistical Analysis

Data are presented as means ± SD. Multiple comparisons between treatments were performed with a one-way ANOVA and the post-hoc Dunnett’s test using GraphPad Prism 6.0 (La Jolla, CA). Results from treatment groups are compared to unexposed control cells. A p-value smaller than 0.05 is considered statistically significant.

## Results

### Localization of BDNF Receptors in Placental Tissues

**Figure 1:**
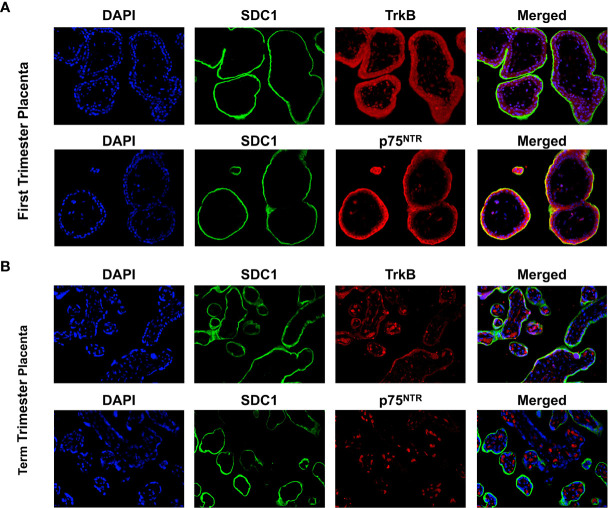
Expression of BDNF receptors in human placental tissues. **(A)** Representative confocal immunofluorescence staining images of showing DAPI (blue) and SDC1 (a syncytiotrophoblast biomarker, green) along with TrkB (red) and p75^NTR^ (red) receptors in the first trimester placenta. **(B)** Confocal immunofluorescence staining images showing DAPI, SDC1, TrkB, and p75^NTR^ in the term placenta.

Syndican-1 (SDC-1), a biomarker for syncytiotrophoblast (STB) was stained green on their epical membrane, TrkB and p75^NTR^ receptors were stained red, and nuclei were stained blue (**Figure 1**). In the first trimester villous placenta, both TrkB and p75^NTR^ staining were most pronounced in the STB and cytotrophoblast cells (CTBs) (**Figure 1A**). Staining was more intense in the STB compared to the CTBs for the p75^NTR^. Spot staining was observed in placental villous stroma tissue for the p75^NTR^ and TrkB.

In term villous placenta, TrkB and p75^NTR^ receptors were primarily observed in the placental villous stroma distinguished it from the first trimester placenta (**Figure 1B**). TrkB receptor staining in STB was reduced, while p75^NTR^ receptor staining was negative. Given these results and our focus on trophoblast cells, TrkB receptors in BeWo cells were examined further in this study.

### Localization and Expression of TrkB in BeWo Cells

**Figure 2:**
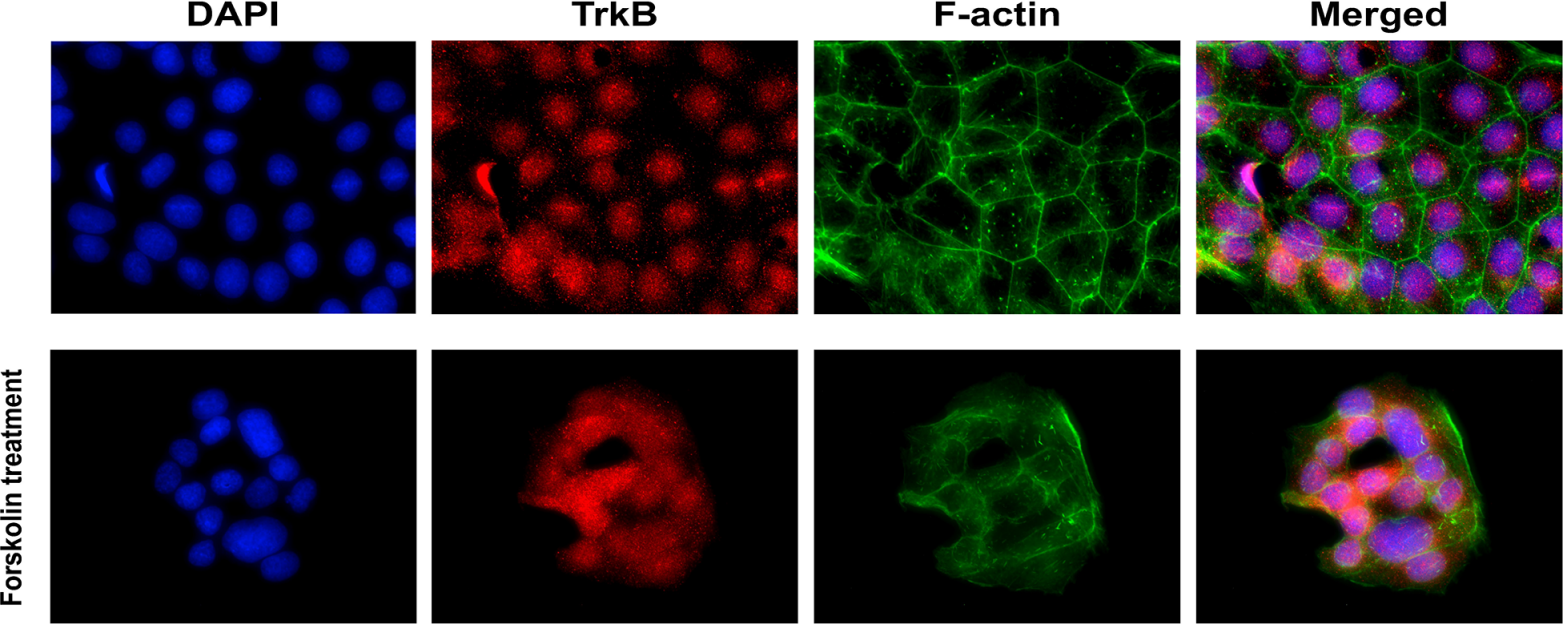
Localization of BDNF receptors in placental BeWo cells. Representative immunofluorescent images showing DAPI, TrkB, and f-actin.

The presence of TrkB receptors in placental BeWo cells was evaluated through immunofluorescence staining. BeWo cells were cultured with and without forskolin treatments. Forskolin treatment was administered to induce fusion of the cells, modeling the villous trophoblast syncytialization (63). F-actin staining distinguished individual BeWo cells before forskolin treatment and diminished in fused cells after treatment. TrkB receptors appeared to be localized in the nuclei, and some appeared to be translocated into the perinuclear region after forskolin-induced fusion. Representative images of TrkB staining in BeWo cells are presented in **Figure 2**.

### PFAS-Induced Disruption of the BDNF Pathway in BeWo Cells

**Figure 3:**
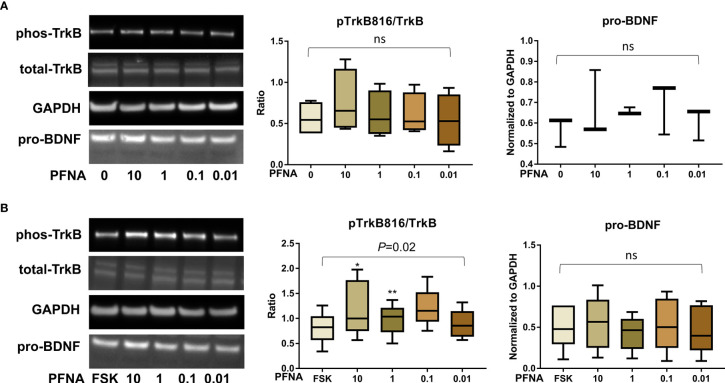
Altered TrkB phosphorylation at high concentrations of PFNA. **(A)** Representative Western blot images of phos-TrkB, total-TrkB, GAPDH, and pro-BDNF in BeWo cells without forskolin (FSK) treatment exposed to PFNA (0, 0.01, 0.1, 1, and 10 µM) and box plots for densitometry analysis; **(B)** Representative Western blot images of phos-TrkB, total-TrkB, GAPDH, and pro-BDNF in BeWo cells with FSK treatment exposed to PFNA (0, 0.01, 0.1, 1, and 10 µM) and box plots for densitometry analysis. ns indicates differences in protein levels were not significant. pTrkB816 represents for phosphorylation of TrkB at Tyr816 residue. *P < 0.05 **P < 0.01 compared to unexposed forskolin-treated cells.

After confirming the presence of TrkB receptors, we examined the effects of PFAS exposure on BDNF production and the ratio of phosphorylated TrkB to total TrkB in BeWo cells with and without forskolin treatment by Western blots. First, the MTS cell viability assay confirmed that the doses used in this study did not cause cell death. Next, we screened the phosphorylation sites, Tyr516, Tyr706/707, and Tyr816 of TrkB receptors in BeWo cells. The phospho-TrkB (Tyr816) was widely detected in these cells with and without forskolin treatment. Finally, Western blot results demonstrated that exposure to PFOS, PFOA, PFBS, and the six-PFAS mixture did not significantly alter BDNF production or activation of TrkB, manifested by the constant ratio of phospho-TrkB (Tyr816) to total TrkB at any given dose (**Figures 3** **–** **7**). Although BDNF production was not changed by PFNA exposures, the ratio of phospho-TrkB (Tyr816) to total TrkB was significantly higher in forskolin-treated BeWo cells exposed to 10 μM and 1 μM of PFNA than in unexposed forskolin-treated BeWo cells (*P*=0.02; **Figure 3**). The same trend was observed in BeWo cells without forskolin treatment but was not significant (**Figure 3**).

**Figure 4:**
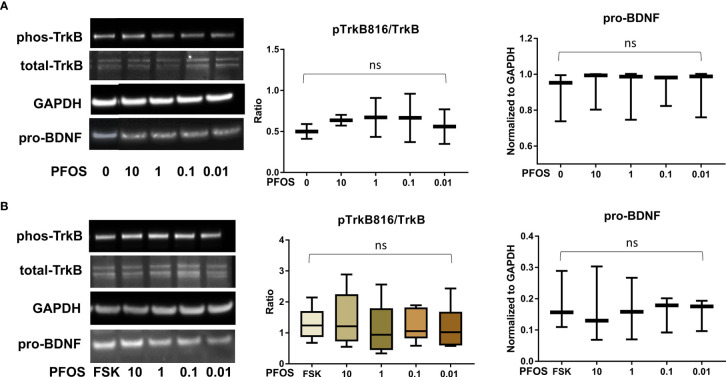
﻿ PFOS exposure did not significantly disrupt the BDNF-TrkB pathway. **(A)** Representative Western blot images of phos-TrkB, total-TrkB, GAPDH, and pro-BDNF in BeWo cells without forskolin (FSK) treatment exposed to PFOS (0, 0.01, 0.1, 1, and 10 µM) and box plots for densitometry analysis; **(B)** Representative Western blot images of phos-TrkB, total-TrkB, GAPDH, and pro-BDNF in BeWo cells with FSK treatment exposed to PFOS (0, 0.01, 0.1, 1, and 10 µM) and box plots for densitometry analysis. ns indicates differences in protein levels were not significant. pTrkB816 represents for phosphorylation of TrkB at Tyr816 residue.

**Figure 5:**
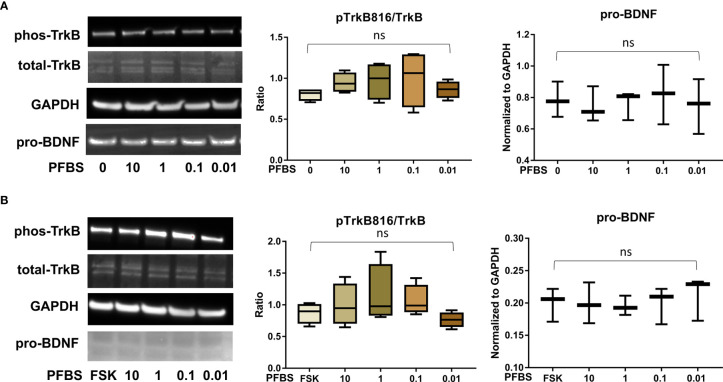
﻿PFBS exposure did not significantly disrupt the BDNF-TrkB pathway. **(A)** Representative Western blot images of phos-TrkB, total-TrkB, GAPDH, and pro-BDNF in BeWo cells without forskolin (FSK) treatment exposed to PFBS (0, 0.01, 0.1, 1, and 10 µM) and box plots for densitometry analysis; **(B)** Representative Western blot images of phos-TrkB, total-TrkB, GAPDH, and pro-BDNF in BeWo cells with FSK treatment exposed to PFBS (0, 0.01, 0.1, 1, and 10 µM) and box plots for densitometry analysis. ns indicates differences in protein levels were not significant. pTrkB816 represents for phosphorylation of TrkB at Tyr816 residue.

**Figure 6:**
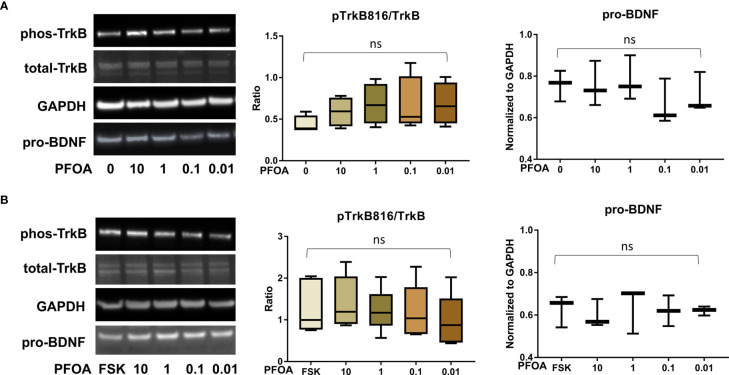
﻿PFOA exposure did not significantly disrupt the BDNF-TrkB pathway. **(A)** Representative Western blot images of phos-TrkB, total-TrkB, GAPDH, and pro-BDNF in BeWo cells without forskolin (FSK) treatment exposed to PFOA (0, 0.01, 0.1, 1, and 10 µM) and box plots for densitometry analysis; **(B)** Representative Western blot images of phos-TrkB, total-TrkB, GAPDH, and pro-BDNF in BeWo cells with FSK treatment exposed to PFOA (0, 0.01, 0.1, 1, and 10 µM) and box plots for densitometry analysis. ns indicates differences in protein levels were not significant. pTrkB816 represents for phosphorylation of TrkB at Tyr816 residue.

**Figure 7:**
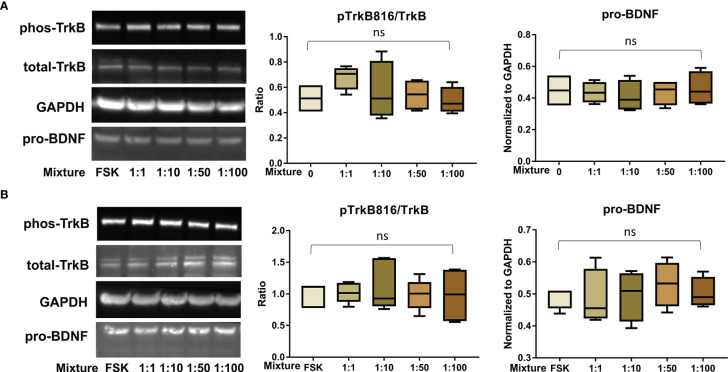
﻿PFAS mixture exposure (detailed in **Table 1**) did not significantly disrupt the BDNF-TrkB pathway. **(A)** Representative Western blot images of phos-TrkB, total-TrkB, GAPDH, and pro-BDNF in BeWo cells without forskolin (FSK) treatment exposed to mixture dilutions (0, 1:1, 1:10, 1:50, 1:100) and box plots for densitometry analysis; **(B)** Representative Western blot images of phos-TrkB, total-TrkB, GAPDH, and pro-BDNF in BeWo cells with FSK treatment exposed to mixture dilutions (0, 1:1, 1:10, 1:50, 1:100) and box plots for densitometry analysis. ns indicates differences in protein levels were not significant. pTrkB816 represents for phosphorylation of TrkB at Tyr816 residue.

## Discussion

This study investigates the BDNF signaling pathway in placental cells and how it is affected by exposure to various PFAS compounds and a PFAS mixture mimicking residents’ blood levels in Pittsboro, North Carolina, USA. Confocal immunofluorescence staining results show that TrkB receptors are present in trophoblasts in the first trimester and term placental villi. Similar expression of p75^NTR^ receptors was observed in first trimester placental tissues. At term, however, p75^NTR^ receptors were primarily located in the placental villous stroma. Immunofluorescence staining results reveal that TrkB receptors are localized to the nuclear and perinuclear regions in BeWo cells. Western blot results indicate that TrkB receptors are activated in BeWo cells by phosphorylation at the Tyr816 site. Additionally, Western blots demonstrate that PFNA exposure at 1 μM and 10 μM doses increased the ratio of phospho-TrkB (Tyr816) to total TrkB. Exposure to other PFAS compounds (PFOS, PFOA, PFBS) and the PFAS mixture did not significantly alter BDNF signaling, as evidenced by unchanged levels of BDNF and phospho-TrkB. Thus, the impacts of PFAS exposure on fetal neurodevelopment previously observed in cohort studies (14–23) do not necessarily occur *via* the placental BDNF pathway.

Although BDNF signaling has been extensively studied in neuronal tissues and cells, little is known about this pathway in gestational tissues. The current study further characterizes the BDNF pathway in placental tissues. BDNF is first synthesized as a precursor protein, prepro-BDNF, which is converted into pro-BDNF by removal of the signal peptide (64). Once the protein is secreted, pro-BDNF is converted to mature BDNF (mBDNF) through proteolytic cleavage by furin and other proprotein convertases (65). We were able to detect pro-BDNF by Western blots in BeWo cell lysates. However, mBDNF levels were close to the limits of detection in BeWo cell culture media, which could be due to limited pro-BDNF secretion or cleavage in our current culture condition. mBDNF is preferentially released *via* a tightly controlled pathway driven by activity-dependent depolarization and Ca^2+^ entry (66). BDNF binds to specific receptors and regulates distinct biological functions. The BDNF receptors in this study were localized in discrete placental domains, indicating that BDNF exhibits distinct functions in different placental cells. BDNF binding to TrkB results in TrkB activation and autophosphorylation at Tyr sites (67). The activated TrkB receptors can induce an array of intracellular signaling cascades (43). TrkB receptor activation at the Tyr490 and Tyr515 residues regulates MAPK/ERK kinases and the PI3K/Akt pathway (68). Both of these pathways activate transcription factors (CREB and C-myc) which trigger neurotrophic functions of cell survival, growth, and differentiation (68). Phosphorylation of TrkB receptors at the Tyr816 site activates phospholipase C γ (PLC γ) (68, 69). The phosphorylation of Tyr706/707 sites can lead to the transphosphorylation of Tyr515 and Tyr816 residues (69, 70). These signaling pathways support many aspects of growth and development such as cell survival, synaptic structure, and synaptic plasticity (43, 59). In BeWo cell cultures, the phosphorylation of the TrkB receptor at its Tyr816 residue was robustly detected, while its Tyr515 and Tyr707 residues were seldom observed.

Expression of neurotrophins like BDNF is susceptible to change in response to perturbations in the maternal environment (40). Exposure to environmental pollutants is a maternal challenge that may affect BDNF expression and signaling in offspring. In rodents, prenatal stress can lead to decreased BDNF expression in offspring from weaning to adulthood (54). Moreover, mouse prenatal exposure to synthetic organic compounds such as bisphenol A (BPA) can induce lasting DNA methylations in transcriptionally relevant BDNF regions (54). Other studies have observed altered cord blood concentrations in humans following acute BPA exposure (35). Given these previous findings, the continued investigation of PFAS-induced BDNF pathway disruptions is imperative. Li et al. found increased TrkB expression in response to 10 μM doses of PFOS in human neuroblastoma cells (58). However, this observation was a proposed compensatory response to a simultaneous PFOS-related decrease in BDNF protein levels, which was seen in other reports (58, 59). Generally, the PFAS mixture and individual PFAS compounds did not significantly regulate the BDNF pathway in BeWo cells in this study. The phosphorylation of TrkB at the Tyr816 site was only increased by PFNA exposure at 1 and 10 μM levels. We speculate that the activation of TrkB receptors is a secondary effect of PFNA exposure on these cells because the protein levels of pro-BDNF were not altered by PFNA exposure and mBDNF was not detected. Further, the activation of TrkB receptors can be triggered by other pathways or ligands unrelated to BDNF (71, 72). Additional studies are needed to investigate the mechanism by which PFNA induces changes in the BDNF signaling pathway and what other neurohormonal pathways could be disrupted by PFAS in both the placenta and fetal brain.

The present study has several limitations. First, the PFAS mixture in this study was based on blood concentrations of Pittsboro residents who were not pregnant. Maternal and placental blood levels of PFAS are unknown at this time. Second, the timeline and dosing allowed us to study the effects of acute exposures but preclude us from drawing conclusions about chronic low-level exposure. Third, our findings are limited to *in vitro* observations. However, we found a strong correlation between *in vitro* (BeWo cells) and *in vivo* (mice placenta and fetal brain tissues) observations of the BDNF pathway upon PM2.5 exposure [unpublished data]. Finally, our findings are based on a single cell model due to the limited availability of STB cell models. JAR, JEG-3—other *in vitro* models of the trophoblast barrier—were unsuitable for this study because they fail to undergo substantial syncytial fusion (73, 74).

This study is the first to localize p75 and TrkB receptors in the human placenta at different stages of gestation and in placental cells originating from choriocarcinoma (BeWo cells). Three individually tested PFAS compounds (PFOA, PFOS, PFBS) and a six-PFAS mixture mimicking residential exposure in Pittsboro, North Carolina, did not induce increased TrkB phosphorylation or alter pro-BDNF levels. Additionally, our results showed that in cells exposed to high concentrations of PFNA, phosphorylation of TrkB receptors increased while pro-BDNF levels remained stable. Although BDNF plays a critical role in brain development throughout all stages of life, it is unlikely to be primarily responsible for the observed neurodevelopmental consequences associated with *in utero* PFAS exposure. Additional investigation is required to understand whether adverse effects arise *via* an alternative placental pathway or are enacted directly on the fetus. These findings contribute to an improved understanding of the understudied BDNF signaling pathway in the gestational tissues and how the pathway is altered by PFAS exposure.

## Data Availability Statement

The raw data supporting the conclusions of this article will be made available by the authors, without undue reservation.

## Ethics Statement

The studies involving human participants were reviewed and approved by the Ethics Committees of Xinhua Hospital affiliated with Shanghai Jiao Tong University School of Medicine and the Duke University Institutional Review Board. The patients/participants provided their written informed consent to participate in this study.

## Author Contributions

MM drafted the manuscript. SL performed research and data analysis. JZ contributed to the study design and drafting the manuscript. LF designed research, performed experiments and data analysis, and revised the manuscript. All authors contributed to the article and approved the submitted version.

## Funding

This work was supported by the Fogarty International Center of the National Institutes of Health under Award Number 1K01TW010828-01 (PI: LF). The funder had no role in study design, data collection and analysis, decision to publish, or preparation of the manuscript.

## Conflict of Interest

The authors declare that the research was conducted in the absence of any commercial or financial relationships that could be construed as a potential conflict of interest.

## Publisher’s Note

All claims expressed in this article are solely those of the authors and do not necessarily represent those of their affiliated organizations, or those of the publisher, the editors and the reviewers. Any product that may be evaluated in this article, or claim that may be made by its manufacturer, is not guaranteed or endorsed by the publisher.

## Footnotes

1
https://www.northcarolinahealthnews.org/2019/07/30/pfas-shows-up-in-haw-river-pittsboro-water-but-little-local-outcry/

2
https://www.northcarolinahealthnews.org/2020/10/29/duke-study-finds-high-pfas-levels-in-pittsboro-residents-blood/

3
https://sites.nicholas.duke.edu/pfas/pfas-research-in-pittsboro-nc/pfas-study-results/
